# Phosphate—latest news and ongoing trials

**DOI:** 10.1093/ckj/sfag108

**Published:** 2026-04-06

**Authors:** Arti Sharma Parpia, Ruchi Kumra, Caleb Mansell, Ziv Harel, Jeffrey Perl, Orit Kliuk Ben-Bassat, Ron Wald

**Affiliations:** Division of Nephrology, St. Michael’s Hospital, Toronto, ON, Canada; Li Ka Shing Knowledge Institute, St. Michael’s Hospital, Toronto, ON, Canada; College of Pharmacy, University of Manitoba, Winnipeg, MB, Canada; Department of Pharmacy, St. Boniface Hospital, Winnipeg, MB, Canada; Faculty of Arts and Sciences, Washington University St. Louis, St. Louis, MO, USA; Division of Nephrology, St. Michael’s Hospital, Toronto, ON, Canada; Li Ka Shing Knowledge Institute, St. Michael’s Hospital, Toronto, ON, Canada; Division of Nephrology, St. Michael’s Hospital, Toronto, ON, Canada; Li Ka Shing Knowledge Institute, St. Michael’s Hospital, Toronto, ON, Canada; Department of Nephrology and Hypertension, Tel Aviv Sourasky Medical Center, Tel Aviv, Israel; Division of Nephrology, St. Michael’s Hospital, Toronto, ON, Canada; Li Ka Shing Knowledge Institute, St. Michael’s Hospital, Toronto, ON, Canada; Department of Nephrology and Hypertension, Tel Aviv Sourasky Medical Center, Tel Aviv, Israel

**Keywords:** chronic hemodialysis, clinical trial, peritoneal dialysis, phosphate binder, phosphatemia

## Abstract

Hyperphosphatemia is present in nearly all patients with kidney failure who receive maintenance dialysis. The direct toxicity of elevated phosphate concentrations had been inferred from basic experimentation and large epidemiologic studies. This has influenced clinical practice in which great efforts are expended in normalizing serum phosphate concentration. Targeted dietary strategies, optimization of dialysis and phosphate-lowering medications are all effective at lowering serum phosphate levels yet each pose challenges for patients and clinicians. The effect of these interventions, individually and collectively, on patient-centered clinical outcomes is also unclear. We will review the evidence from clinical trials for each of these measures and discuss ongoing research that is testing optimal targets for serum phosphate.

## PHOSPHATE—LATEST NEWS AND ONGOING TRIALS

Hyperphosphatemia is nearly ubiquitous among patients with kidney failure who receive maintenance dialysis [1]. Dietary phosphate intake routinely exceeds excretion due to absent or limited endogenous kidney function and the limitations of conventional dialysis modalities in removing phosphate [2]. Experimental data have implicated hyperphosphatemia as a mediator of the vascular calcification that is common in patients with kidney failure [3, 4]. This may explain why epidemiologic studies have consistently demonstrated an association between hyperphosphatemia and death [5]. In addition, hyperphosphatemia is associated with secondary hyperparathyroidism, which may further exacerbate hyperphosphatemia through the increased phosphate release from the bone [6]. Hyperphosphatemia has classically been associated with uremic pruritus but this relationship has recently been questioned [7]. The perceived toxicity of hyperphosphatemia has entrenched the lowering or normalization of serum phosphate concentration as a fundamental element in the care of patients with kidney failure [8, 9].

This review will discuss the core components of phosphate control in maintenance dialysis recipients: dietary restriction, dialysis-based removal, and phosphate-lowering medications, with a preferential focus on data from clinical trials. We will also discuss ongoing research examining optimal phosphate targets. The physiology of phosphate metabolism [10] andA the putative toxicity attributed to phosphate in patients with kidney disease [11, 12] have been extensively delineated and are outside the scope of this review. We will deliberately refer to the phosphate anion (PO_4_^−3^) as the prevalent form on phosphorus in the body though where relevant, especially in dietary studies, we will specify the phosphorus element.

## DIET

Dietary phosphate restriction has long been considered a cornerstone of the management of hyperphosphatemia in patients with kidney failure. Dietary interventions are perceived as simple and economical but may present several challenges to patients [13].

Early clinical guidelines focused on limiting dietary intake of elemental phosphorus to 800–1000 mg per day, with a focus on restricting dairy, animal protein, nuts, and legumes [14]. More recent guidelines recommend that patients limit their intake of dietary phosphorus to treat persistent hyperphosphatemia, but refrain from suggesting a specific daily intake ceiling; instead, they emphasize individualizing treatment based on patient needs, clinical judgement, and considering the source of dietary phosphorus [15]. Various targeted dietary interventions have shown efficacy in lowering serum phosphate concentration.

### A focus on protein

Dietary protein is a significant source of phosphate and protein restriction has been shown to be efficacious in lowering serum phosphate concentration in patients with advanced chronic kidney disease (CKD) not yet requiring dialysis; however, this strategy is not recommended in patients receiving dialysis due to their increased protein requirements and the risk of protein energy wasting from inadequate protein consumption [16, 17]. One strategy involves administering high-protein meals in conjunction with a phosphate binder to help patients meet their protein requirements while maintaining serum phosphate control. This approach was tested in a trial of 110 hemodialysis (HD) recipients with hypoalbuminemia. Participants were randomized to receive a high-protein–phosphate-rich meal (50–55 g protein, 400–500 mg phosphorus) plus lanthanum carbonate or a low-protein–low-phosphate meal (<1 g protein, <20 mg phosphorus) during HD [18]. The authors demonstrated that high-protein meals coadministered with lanthanum increased serum albumin, a surrogate for nutritional status, without raising the serum phosphate concentration.

Choosing foods with a lower phosphorus to protein ratio can also be efficacious in helping dialysis recipients meet their protein needs without increasing the serum phosphate concentration. Egg whites have the lowest phosphorus to protein ratio among animal proteins, with almost 4 g of protein and a negligible amount of phosphorus per egg white. Two randomized controlled trials have demonstrated that replacing meat or fish with egg whites can effectively reduce the serum phosphate concentration in HD recipients while maintaining adequate protein intake. Guida *et al*. (*n* = 23) instructed patients to replace one serving of fish or meat with five to six egg whites in three meals per week for 3 months, resulting in significantly lower serum phosphate concentrations compared to usual diet (1.58 ± 0.32 vs. 2.13 ± 0.26 mmol/l; *P* < .001) with no changes in albumin or body weight [19]. Similarly, Azmandian *et al*. (*n* = 150) implemented an 8-week intervention in which three meat-based meals per week were replaced with six egg whites each, which also significantly reduced serum phosphate (1.45 ± 0.33 vs. 2.16 ± 0.48 mmol/l; *P* = .001) and raised serum albumin levels (45 ± 7 vs. 37 ± 4 g/l; *P* = .001) as compared to a standard low-phosphorus diet [20]. Both studies support egg whites as a high-quality, low-phosphorus protein source that can help HD recipients meet nutritional requirements without elevating the serum phosphate concentration.

### Minimizing consumption of phosphate-containing additives

Phosphate-containing additives are increasingly found in processed and fast foods, and tend to have higher bioaccessibility and bioavailability (>60% intestinal absorption), compared to plant-based foods (30%–40%) [21, 22]. As a result, it is recommended that the bioavailability of phosphate (e.g. animal, plant, and additives) be considered when providing dietary interventions to address hyperphosphatemia [9, 15].

Sullivan *et al*. conducted a trial of 279 HD recipients with hyperphosphatemia that compared receipt of education that focused on the identification and avoidance of phosphate additives to usual care [23]. Patients randomized to the education tool exhibited a modest (∼0.20 mmol/l) decline in serum phosphate concentrations after 3 months. Similarly, de Fornasari *et al*. randomized 134 patients with hyperphosphatemia, where those allocated to the intervention group were counselled to replace processed food containing phosphate additives with similar additive-free foods versus routine nutritional counseling in the control group [24]. After 3 months of follow-up, those allocated to the intervention group had a significant reduction in serum phosphate (from 2.33 ± 0.45 to 1.62 ± 0.52 mmol/l, *P* < .001), while the control group showed no significant change.

### Plant-based proteins

Several dietary strategies incorporating plant-based proteins, which have lower intestinal phosphate bioavailability, have demonstrated effectiveness in the management of hyperphosphatemia. Byrne *et al*. conducted a pilot randomized trial in 74 HD recipients to evaluate the safety and effectiveness of incorporating plant-based proteins, which have historically been excluded from conventional low-phosphorus diets [25]. Participants were assigned to either a standard low-phosphorus diet or a modified diet in which two daily servings of animal protein (7 g protein/serving) were replaced with two servings comprising legumes or nuts, while avoiding phosphate additives and encouraging whole grains. Despite similar dietary phosphate intake, the modified phosphate diet achieved comparable serum phosphate control, along with higher dietary fiber intake and greater food variety, compared to the standard diet, supporting the safe inclusion of nuts, legumes, and whole grains as part of a low-phosphorus diet.

A Cochrane systematic review determined, with very-low-quality evidence, that low-phosphorus diets decreased serum phosphate concentrations compared to standard diets (mean difference: -0.18 mmol/l) [26]. Similarly, a pooled analysis comprising 11 trials demonstrated that monthly dietary interventions significantly reduced serum phosphate levels (mean difference: −0.28 mmol/l) versus comparator diets [27]. Despite notable heterogeneity among studies and a low certainty of evidence, the authors concluded that consistent dietary advice is both effective and safe in lowering serum phosphate without adversely affecting nutritional status.

**Figure 1:**
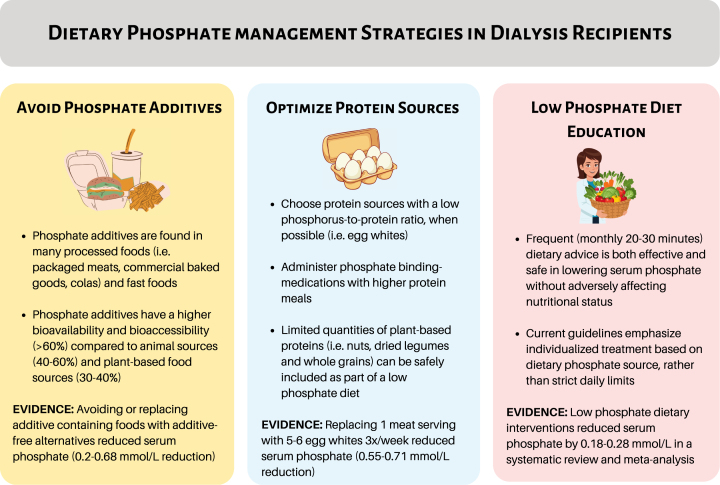
Overview of dietary strategies for phosphate lowering.

Overall, dietary interventions are effective in achieving modest reductions in serum phosphate concentration (Fig. 1). However, the impact of these strategies on hard clinical outcomes such as mortality and CKD progression remains uncertain. Noting the centrality of food enjoyment to patients, future trials that modify dietary phosphorus intake must consider the impact of diet on quality of life.

## DIALYSIS-BASED STRATEGIES TO LOWER SERUM PHOSPHATE CONCENTRATION

A typical 4-h HD session will remove ∼1000 mg of phosphate [28]. Even if patients are adherent to a traditional phosphate-restricted diet of 1000 mg per day, a conventional thrice weekly HD regimen will fall far short of maintaining balance. Notably, incremental HD regimens (i.e. twice weekly HD sessions instead of the conventional thrice weekly regimen) that are increasingly gaining traction may further compromise extracorporeal phosphate removal especially as residual kidney function diminishes. In addition, high-flux large surface area dialyzers, an optimal vascular access, and maximization of blood flow, are vital but not sufficient to achieving optimal phosphate clearance.

### Intensified dialysis schedules

Phosphate removal is exquisitely sensitive to time on therapy [29, 30]. Though it has a small molecular weight, phosphate removal is highest in the first hours of HD reflecting removal from the plasma space. Phosphate clearance then plateaus as phosphate is mobilized to the plasma from other body compartments. As a result, phosphate clearance mirrors that of larger-sized “middle molecules.” Dialysis regimens that maximize time on therapy, including short daily HD [31], in-center nocturnal HD [32–34], or home nocturnal HD [31, 35], have conferred a reduction in the serum phosphate concentration. Prolonged dialysis regimens enable a reduction in the burden of phosphate-lowering agents and in some cases, there is a need to *add* phosphate to the dialysate [31, 35].

### Hemodiafiltration

The added convective clearance conferred by hemodiafiltration (HDF) above conventional high-efficiency high-flux HD may enhance phosphate removal [36, 37]. A recent meta-analysis demonstrated that HDF conferred a lower predialysis serum phosphate concentration of ∼0.3 mmol/l as compared to high-flux HD but there was a great deal of heterogeneity among included trials (*i*^2^ = 78%) [38]. One randomized controlled trial demonstrated that HDF removed ∼200 mmol more phosphate than a conventional high-flux HD session (1099 ± 239 vs. 864 ± 366 mmol/session; *P* < .05) [39]. It should be noted that even if HDF enhances phosphate removal, this may not translate into a difference in predialysis serum phosphate concentration as the latter will be affected by a variety of co-interventions (e.g. diet, binder use, use of activated vitamin D, and calcimimetics). It is notable that in the comparison of high-dose hemodiafiltration with high-flux hemodialysis (CONVINCE) trial, which demonstrated a survival benefit with HDF, predialysis phosphate concentrations were comparable in the HDF and high-flux HD arms over the trial’s follow-up period [40].

### Issues unique to peritoneal dialysis

Maintenance of residual kidney function is a major contributor to optimal phosphate control in patients receiving peritoneal dialysis (PD) [41]. Observational data suggest that peritoneal membrane solute transfer characteristics may influence peritoneal phosphate clearance with higher clearance generally observed among those with faster peritoneal solute transfer [42,43]. With regard to modality, continuous ambulatory peritoneal dialysis (CAPD) may yield superior clearance compared to automated regimens particularly in patients with low peritoneal solute transfer rates [42]. In a large prospective cohort study from Brazil that examined PD modality changes, CAPD was associated with lower serum phosphate concentrations as compared to automated PD (−0.22 ± 0.54 mmol/l) [44]. These findings suggest that longer dwell times confer better phosphate clearance.

## PHOSPHATE-LOWERING MEDICATIONS

Most phosphate-lowering agents operate on the premise of binding phosphate in the gastrointenstinal (GI) tract leading to the excretion of the binder–phosphate complex in the feces. Consumption of binders concurrent with meals maximizes their ability to bind dietary phosphate. Table 1 summarizes features of phosphate-lowering agents that are in common use including information on their relative phosphate-binding capacity [45].

**Table 1:** Summary of commonly used phosphate-lowering agents.

Phosphate-lowering medication	Phosphate binder equivalent dose*	Dosage	Advantages	Shortcomings
Calcium carbonate	1.00 (1000 mg calcium carbonate tablet = 400 mg elemental calcium)	Start at 300–400 mg elemental calcium 3×/day with meals. Do not exceed 1500 mg elemental calcium/day.^c^	Relatively inexpensive, widely available, and available in pill and chewable forms.	Hypercalcemia, constipation, and nausea.
Calcium acetate	0.67^a^ (667 mg calcium acetate tablet = 166 mg elemental calcium)	Start at two tablets 3×/day with meals. Most patients require three to four tablets with each meal.^d^	Relatively inexpensive and widely available.	Hypercalcemia and nausea.
Lanthanum carbonate	1.00^a^ (500 mg lanthanum carbonate tablet)	Start at 250–500 mg 3×/day with meals.^e^ Maximum dosing up to 4500 mg/day has been studied.^f^ Must be chewed or crushed.	Reduced pill burden and effective in wide pH ranges.	GI side effects and more expensive than calcium-based binders.
Sevelamer hydrochloride/sevelamer carbonate	0.60^a^ (800 mg sevelamer tablet)	Start at 800 mg 3×/day with meals. Maximum dose 7200 mg/day.^g^	Calcium free and may reduce LDL.	GI side effects and more expensive than calcium-based binders.
Sucroferric oxyhydroxide	1.4^b^ (2.5 g sucroferric oxyhydroxide tablet = 500 mg iron)	Start at 500 mg iron 3×/day with meals. Maximum dose 3000 mg iron/day.^h^	Calcium free, lower pill burden, works in wide pH ranges, and increases iron stores.	Diarrhea, nausea, discoloration of stool, and more expensive than calcium-based binders.
Tenapanor	Not applicable. Inhibits paracellular phosphate absorption.	30 mg 2x/day. May reduce dose based on serum phosphate and GI tolerability.^i^	Minimally absorbed, reduced pill burden, and effective adjunct to binders.	Diarrhea and expense.

*Phosphate binder equivalent dose expressed in reference of 1000 mg of calcium carbonate.

Adapted from Jadav *et al*. [47].

References:

a Daugirdas *et al*. [45].

b Schoer MB and Coyne DW. *Mineral and Bone Disease in Handbook of Dialysis*, 6th edn. Wolters Kluwer, 2026.

c Lexicomp. *Calcium carbonate*. Wolters Kluwer Health. (21 December 2025, date last accessed).

d Cipla USA, Inc. *Calcium acetate capsules, USP 667 mg: prescribing information*. Cipla USA, Inc. (June 2021, date last accessed).

e Wazny L, Kawchuk K, Hingwala J. Chronic kidney disease. In: Canadian Pharmacists Association (ed.), *Therapeutic Choices*. 2021 ed. Ottawa (ON): Canadian Pharmacists Association, 2021, 1777–93.

f Shire Pharma Canada ULC. Fosrenol® (lanthanum carbonate) product monograph. Shire Pharma Canada ULC, 2016.

g Lexicomp. *Sevelamer carbonate and sevelamer hydrochloride*. Wolters Kluwer Health. (21 December 2025, date last accessed).

h Vifor Fresenius Medical Care Renal Pharma Ltd. *Velphoro® (sucroferric oxyhydroxide) product monograph*. Vifor Fresenius Medical Care Renal Pharma Ltd. (5 January 2018, date last accessed)..

i Lexicomp. *Tenapanor*. Wolters Kluwer Health. (21 December 2025, date last accessed).

The advent of calcium-based binders in the 1980s supplanted the widespread use of aluminum-based binders due to the toxicity of the latter [46]. Calcium-based agents, notably calcium acetate and calcium carbonate, are effective, widely available, and relatively inexpensive. The elemental calcium contents of calcium acetate and calcium carbonate are 25% and 40%, respectively. Calcium carbonate is available in a wider array of tablet sizes (including chewable forms) allowing greater dosing flexibility and a lower pill burden as compared to calcium acetate.

The introduction of noncalcium-based binders was fueled by concerns that calcium-based agents led to positive calcium balance and accelerated vascular calcification, potentially heightening the risk of cardiovascular events [47]. The 2017 Kidney Disease: Improving Global Outcomes (KGIDO) Clinical Practice Guidelines recommended limiting the use of calcium-based binders in all patients, rather than solely in those with recurrent or persistent hypercalcemia as previously advised [9]. Nonetheless, the benefits of noncalcium binders in reducing cardiovascular events remain controversial [47].

Sevelamer, a nonabsorbable cationic polymer, emerged as the first alternative to calcium-based binders. The Treat-to-Goal trial randomized 200 HD recipients to sevelamer hydrochloride or calcium acetate and demonstrated significantly reduced progression of coronary artery calcification (CAC) (CAC score: 36.6 vs. 0; *P* = .03) and less hypercalcemia in patients treated with sevelamer after 1 year of follow-up (16% vs. 5%; *P* = .04) [48]. The Dialysis Clinical Outcomes Revisited (DCOR) trial enrolled 2103 HD recipients to test whether sevelamer would reduce all-cause mortality as compared to calcium-based binders. Sevelamer had no demonstrable effect on mortality (17.7 vs. 17.4 death/100 patient-years; *P* = .8) or cardiovascular events as compared to calcium-based products [49].

Lanthanum is a rare earth element with effective phosphate-binding properties [50, 51]. The Outcome Study of Lanthanum Carbonate Compared With Calcium Carbonate on Cardiovascular Mortality and Morbidity in Patients with Chronic Kidney Disease on Hemodialysis (LANDMARK) trial enrolled 2309 HD patients with hyperphosphatemia who had at least one risk factor for vascular calcification [52]. The trial was conducted at 273 sites in Japan. The participants were randomized to receive either lanthanum carbonate or calcium carbonate in an open-label fashion with the intent of maintaining serum phosphate concentration between 1.13 and 1.94 mmol/l. After a median follow-up of ∼3 years, there was no significant difference in the primary composite outcome of cardiovascular events (Hazard Ratio (HR) 1.11; 95% Confidence Interval (CI) 0.88–1.41, *P* = .37) or the secondary outcome of all-cause mortality (HR 1.10; 95% CI 0.88–1.37, *P* = .42). Notably, the trial did not achieve its target sample size and the event rate of the primary outcome was lower than expected.

In addition to being effective at binding phosphate, ferric citrate and sucroferric oxyhydroxide have the theoretical practical benefit of concomitantly addressing the iron deficiency that is widely prevalent in HD recipients [53–57]. There have been no clinical trials evaluating the relative merits of iron-based binders on hard clinical outcomes. The Evaluate the New Phosphate Iron-Based Binder Sucroferric Oxyhydroxide in Dialysis Patients (EPISODE) trial randomized 115 maintenance HD patients to sucroferric oxyhydroxide or lanthanum carbonate (in the second factor of the trial discussed below, patients were also randomized to strict or standard phosphate control). The percentage change in CAC scores after 1 year, the trial’s primary outcome, did not differ between the two study agents [58].

Tenapanor is a newer agent that inhibits the intestinal Na-H antiporter inducing intracellular acidosis [59]. This leads to conformational changes in cell junction proteins thereby leading to the inhibition of passive paracellular phosphate absorption. A recent meta-analysis showed that tenapanor conferred a mean serum phosphate reduction of 0.45 mmol/l compared to placebo [60].

While all phosphate binders are effective at lowering serum phosphate concentrations, there is no evidence to support an effect on patient-relevant clinical outcomes. Moreover, phosphate binders entail a significant pill burden, with implications for patient quality of life, and are costly [61–63]. A recent Cochrane review concluded that no clinically important benefits of phosphate binders were identified for cardiovascular death or coronary artery calcium score compared to placebo/usual care [64].

## THE INTENSITY OF PHOSPHATE CONTROL: RECENT AND ONGOING TRIALS

There is no agreement on the optimal target for the serum phosphate concentration. While suggesting that the serum phosphate concentration be lowered toward the normal range in maintenance dialysis recipients, the KDIGO Clinical Practice Guidelines acknowledge that this is a weak recommendation based on low-quality evidence [8].

The EPISODE trial examined the effect of strict (serum phosphate 1.13–1.45 mmol/l) versus standard (serum phosphate 1.62–1.94 mmol/l) serum phosphate targets on the change in CAC. At 12 months, the strict phosphate group achieved a mean serum phosphate of 1.51 mmol/l, as compared to 1.79 mmol/l in the standard arm. The change in coronary calcification was modestly, but significantly, reduced in patients randomized to strict phosphate control [CAC progression: 8.52 (IQR 1.0–23.9)% vs. 21.8 (IQR: 10–36.1)%; *P* = .006] [58].

HiLo was a multicenter trial in the USA that tested whether a lower (<1.78 mmol/l) versus higher (≥2.10 mmol/l) phosphate target reduced the hierarchical outcome of all-cause mortality and all-cause hospitalization in HD recipients [65, 66]. The trial was initially launched as a cluster trial and HD centers were allocated in their entirety to a given phosphate strategy, but individual patients still had to consent to receive the strategy to which their center was assigned. After noting important imbalances in intergroup baseline characteristics, the trial shifted to individual level patient consent. The trial was halted after the recruitment of 793 participants (544 in the cluster phase and 249 in the individual-level consent phase) due to slow enrollment and a perception of inadequate intergroup differences in serum phosphate concentration. Though underpowered to answer this question, participants randomized to the lower phosphate target did not experience a reduction in the primary hierarchical outcome as compared to those in the higher target arm (win ratio 0.97; 95% CI 0.55–1.71).

**Figure 2:**
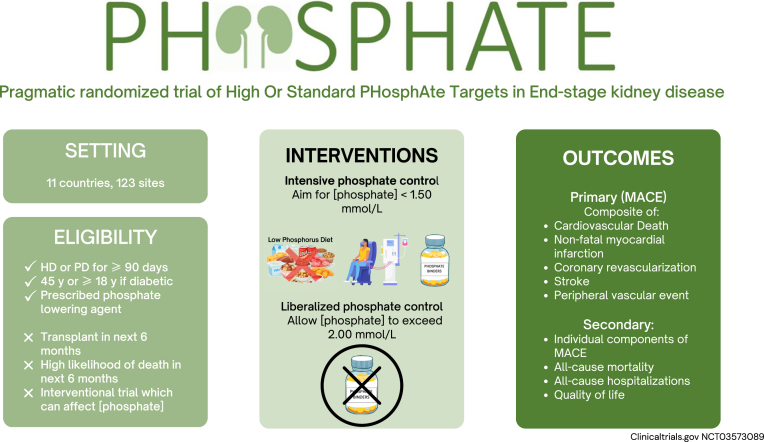
Overview of the PHOSPHATE trial.

The multinational Pragmatic randomised trial of High Or Standard PHosphAte Targets in End-stage kidney disease (PHOSPHATE, NCT03573089) will randomize at least 3600 dialysis patients (HD and PD) to one of two treatment strategies. In the intensive arm (target serum phosphate <1.50 mmol/l), dietary advice, optimization of dialysis, and phosphate-lowering agents will be deployed to aim for serum phosphate normalization. All available phosphate binders may be used alone or in combination to achieve the treatment goal. In the liberalized arm, phosphate binders will be discontinued and dietitians will be instructed not to discuss phosphate restriction with participants. Phosphate binders may be restarted if the serum phosphate concentration surpasses 2.50 mmol/l, which represents a level that many clinicians might consider excessively high. The primary endpoint is the composite of cardiovascular death, nonfatal cardiovascular events (myocardial infarction, stroke, and peripheral arterial events) or coronary revascularization. The secondary outcome measures include individual components of the primary composite endpoint, all-cause death, and utility-based quality of life using the EuroQol-5 Dimension. This trial is anticipated to conclude at the end of 2027. Figure 2 provides an overview of the trial design.

## CONCLUSIONS

Management of hyperphosphatemia remains a core component of the care provided to patients with kidney failure who receive dialysis. Efforts to normalize the serum phosphate concentration, as suggested by current guidelines, require a multifaceted approach including thoughtful dietary strategies, optimization of the dialysis prescription, and the administration of phosphate-lowering agents. Each of these interventions poses unique challenges to patients. While the risks attributed to hyperphosphatemia are widely appreciated, it is still unclear whether the efforts that are invested to lower the serum phosphate concentration translate into better clinical outcomes. Ongoing studies should provide greater clarity on this contentious issue. If a lower serum phosphate concentration is found to be beneficial, this will hopefully spur the development of dietary, dialytic, and pharmacologic strategies that maximize patient health with minimal intrusion on quality of life.

## Data Availability

No new data were generated or analyzed in support of this research.
